# Locally Acquired Dengue in Townsville, Australia, 2024–2025: An Outbreak Report in a Non-Endemic Region with *w*Mel *Wolbachia*-Infected *Aedes aegypti*

**DOI:** 10.3390/tropicalmed11030066

**Published:** 2026-02-26

**Authors:** Kyra Thompson, Scott Lyons, Katherine Malone, Jesse Fryk, Alyssa Pyke, Kate Murton

**Affiliations:** 1 Townsville Public Health Unit, Townsville Hospital and Health Service, 242 Walker Street, Townsville City, QLD 4810, Australiakatherine.malone@health.qld.gov.au (K.M.);; 2 Public Health Virology, Public and Environmental Health Reference Laboratories, Coronial and Public Health Sciences, 39 Kessels Road, Coopers Plains, Brisbane, QLD 4108, Australia

**Keywords:** dengue, *Wolbachia*, outbreak, Queensland, neglected tropical diseases

## Abstract

During the 2024/2025 wet season, Townsville had its first sustained autochthonous outbreak of dengue disease caused by dengue virus type 2 (DENV-2), the second locally transmitted outbreak of dengue since 2014 following the introduction of *w*Mel strain *Wolbachia*-infected mosquitoes, a control strategy for dengue virus (DENV) and other *Aedes*-transmitted arboviruses. In comparison to two recorded locally acquired cases of dengue in 2020, the 2024/2025 outbreak resulted in sixteen cases in two inner-city suburbs of Townsville during the wet season associated with higher-than-average rainfall. This second dengue outbreak since 2014 highlights that Townsville and other north Queensland communities where *Wolbachia* mosquito programs have been deployed remain vulnerable to DENV incursions and local disease outbreaks despite the apparent high coverage of *Wolbachia*-infected mosquitoes. Whilst these control strategies have likely contributed to a reduction in the number and frequency of autochthonous DENV outbreaks in north Queensland, ongoing maintenance and monitoring of *Wolbachia*-infected mosquito coverage is necessary, together with timely review and improvement in dengue awareness and prevention health promotion activities in the community.

## 1. Introduction

Townsville is a major coastal city in North Queensland with a population of 247,234 [1]. This regional city has a dry tropical climate with a distinct wet season from November to April [2]. Dengue virus (DENV) belongs to the *Flaviviridae* family, genus *Orthoflavivirus.* Dengue disease is caused by one of the four antigenically related serotypes (DENV-1, DENV-2, DENV-3 and DENV-4) that is transmitted to humans predominantly by *Aedes aegypti* and *Aedes albopictus* mosquitoes. Both species are indigenous to Queensland, Australia, although *Ae. aegypti* is established in mainland Queensland [3,4]. DENV typically causes a self-limited acute viral syndrome, which may be complicated in severe disease forms such as dengue shock syndrome or haemorrhagic fever [5]. Symptoms can vary depending on disease severity, and can include myalgia, arthralgia, headache, retroorbital pain, to petechiae, mucosal membrane bleeding and hepatomegaly in more severe cases [6]. Dengue is endemic in over 100 countries and is considered by the World Health Organization (WHO) as a significant global health threat [5,6,7]. In Australia, dengue is a nationally notifiable disease, meaning confirmed and probable cases must be reported to State and Territory public health authorities [8], with Queensland using state-specific guidelines for health practitioners and public officials to identify, treat, control and prevent dengue cases [9]. Any cases notified to Queensland Health are entered onto the Queensland Health Notifiable Conditions System.

Following the introduction of DENV into Australia from Mauritius in 1873, dengue epidemics became widespread in eastern Australia—spanning from Thursday Island, Torres Strait to Gosford, New South Wales up to 1955 [10,11]. The epidemiologic pattern since 1955 had shifted with most of eastern Australia no longer experiencing dengue endemicity due to the contraction of the mosquito vector to north Queensland, where episodic outbreaks continued to occur. This was a result of reduced water tank usage, improved sanitation and maintenance of properties, and utilization of insecticides by homeowners [11]. Whilst remaining a DENV endemic-free region, from 2012 to 2022, Australia had a median incidence of 1466 cases per year with the majority being imported cases from endemic countries in Asia and Oceania [12].

Vector control strategies such as targeted residual spraying, larval control and personal protective measures are the mainstay for preventing dengue infection [5]. Since 2011, regions in north Queensland have adopted the *Wolbachia*-conferred viral blocking strategy, using the *w*Mel strain, to control dengue [13,14]. *Wolbachia* is a naturally occurring intracellular bacterium that infects mosquitoes [15], reducing the mosquito’s ability to transmit arboviruses, and modestly shortening the mosquito’s lifespan [5,15]. For susceptible arboviruses including DENV, competition for host cell components that are key for viral RNA replication, protein synthesis and activation of inherent host cellular immune responses can also reduce arboviral replication efficiencies, further blocking viable virus production to levels below that required for efficient transmission to vulnerable human and animal hosts [5]. Inheritance of *Wolbachia* amongst the *Ae aegypti* population is self-sustaining, which has led to a successful mosquito population replacement strategy with *Wolbachia*-infected mosquitoes [5]. In 2014–2017, the *Wolbachia* program was implemented in the Townsville region [16]. Since June 2014, the average frequency of *w*Mel strain *Wolbachia*-infected mosquitoes across the Townsville region has been 93%.

Prior to the introduction of *w*Mel strain *Wolbachia*-infected mosquitoes in the Townsville region, dengue outbreaks occurred almost annually and required significant public health intervention to stop transmission. The last large-scale outbreak occurred in 2014, with a DENV-1 outbreak in Townsville and Charters Towers that resulted in a total of 47 cases [17]. Over 3000 properties were inspected during the response. This outbreak originated from Cairns, where 132 cases were reported. Dengue notifications to the Townsville Public Health Unit (TPHU) since the introduction of *w*Mel strain *Wolbachia*-infected mosquitoes have overwhelmingly been imported cases from returned travelers who had visited endemic countries, primarily from the South East Asia region [18]. Since the introduction of *Wolbachia*, there has only been one other instance of local transmission occurring in 2020 with two locally acquired DENV-1 cases living on adjacent properties. Phylogenetic analysis of retrieved DENV-1 sequences from these two patients indicated they were most closely related to 2020 sequences from the Americas (A. Pyke, unpublished data).

In December 2024 to April 2025, Townsville had its first sustained outbreak of locally acquired dengue since the introduction of *w*Mel strain *Wolbachia*-infected mosquitoes. In the context of the current report, the term ‘sustained outbreak’ is being used to denote that the outbreak was sustained over a significant period of three to four months involving 16 cases and potentially multiple transmission events. This report will describe the outbreak, and the public health and environmental health response, as well as discuss possible factors that may have led to locally acquired cases in a *w*Mel strain *Wolbachia*-infected mosquito setting.

## 2. Materials and Methods

Data were collected from the Queensland Health Notifiable Conditions System (the Queensland Health Integrated Electronic Medical Record (Version 2023.2.0.27)) and Auslab (the Queensland Health Laboratory Information System) and were collated on a Microsoft Excel spreadsheet (Version 2502 (Build 18526.20472 Click-to-Run)). All locally acquired cases notified to Townsville Public Health Unit (TPHU) from 6 January to 20 April 2025 were included in this outbreak. Rainfall data was extracted from the Australian Bureau of Meteorology website from the Townsville Aero station, the weather station closest to the relevant suburbs.

## 3. Results

### 3.1. Description of Outbreak

**Figure 1:**
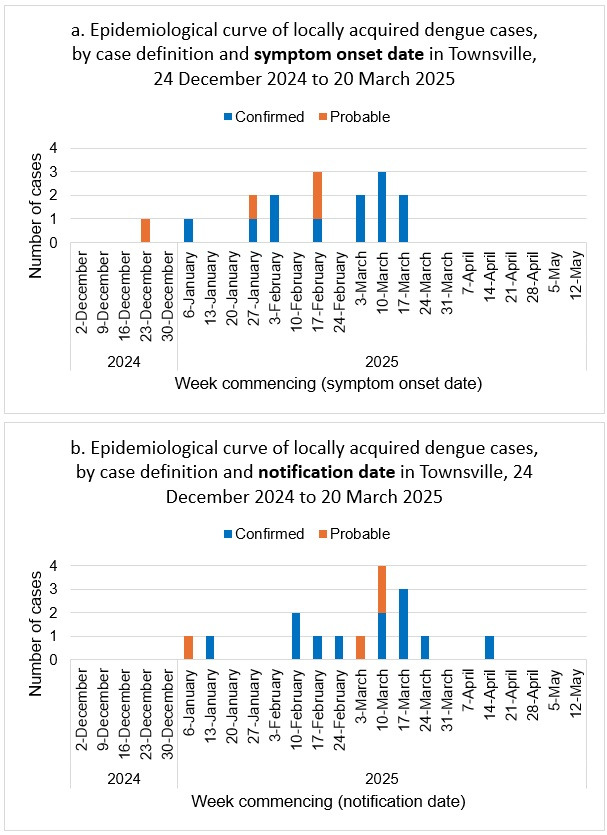
Epidemiological curve of locally acquired dengue cases by (**a**) symptom onset and (**b**) notification date in Townsville, 24 December 2024 to 20 March 2025.

Sixteen cases were identified between December 2024 and April 2025 (epidemiological curve Figure 1; line list in Appendix A). There were twelve confirmed cases based on laboratory and clinical evidence, and four probable cases based on epidemiological and clinical evidence. The case definition for probable and confirmed cases was based on the Queensland Health Dengue Guideline for Public Health Units (Appendix B).

On 7 January 2025, TPHU received notification of a flavivirus (unspecified)-positive case living in the inner-city suburb of North Ward. This patient (Case 1) became symptomatic on Christmas Eve 2024 and delayed presentation and subsequent testing due to travel within Queensland over the Christmas period. This notification was closed as ‘not a case’ because their flavivirus result was unspecified, they had not traveled overseas and there was no epidemiological link. Therefore, this did not meet the case definition for probable or confirmed dengue. On 13 January 2025, TPHU were notified of the first confirmed case (Case 2) with a positive NS1 antigen and DENV PCR, who had no epidemiological link. This person lived less than 100 m from Case 1, meaning that an epidemiological link had been identified and Case 1 could now be classified as a probable case of dengue. Case 1 could not be confirmed by laboratory testing due to their delay in initial testing. The index case was not identified as no known imported cases had epidemiological links to the cases in this outbreak.

The next four cases lived in the suburb of South Townsville, approximately 3 km from the first case in North Ward. These cases were notified between 11 February and 25 February 2025, and their symptom onset was between 6 January and 25 January 2025. Three of these cases lived in or visited the same household and the fourth lived next door. There have been no identified links between the North Ward and South Townsville cases.

Between 3 March and 14 April 2025, the remaining ten cases in North Ward were notified to TPHU. These cases lived within 120 to 200 m of the first two North Ward cases. Six of these cases had either lived at or visited one house (House X) and three cases lived in properties adjacent to House X. Three of these cases were classified as probable based on the epidemiological link and their clinical manifestations.

The median age of the cases was 50 years (range of 25 to 86 years), and thirteen cases were male. Two individuals identified as Aboriginal, and one individual identified as both Aboriginal and Torres Strait Islander. All cases made a recovery from their viraemia and none were classified as severe dengue or dengue with warning signs [6]. Two cases were hospitalized; however, they required admission for reasons other than dengue fever.

**Figure 2:**
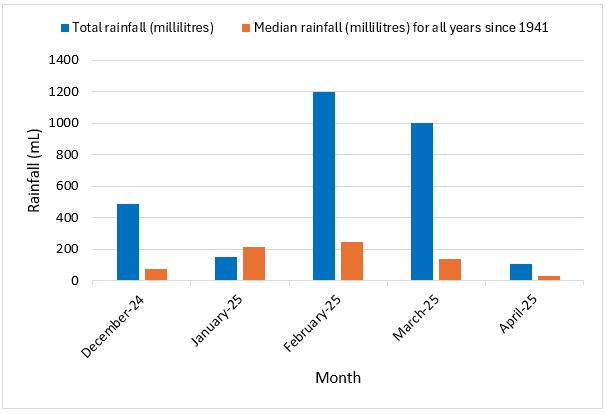
Townsville rainfall (Townsville Aero station) by month, December 2024 to April 2025 [19].

The total rainfall in Townsville for the months of December in 2024 and February to April in 2025 was considerably higher compared to the median total rainfall for the respective months between 1941 and 2024 (Figure 2).

### 3.2. Laboratory and Environmental Investigations

**Figure 3:**
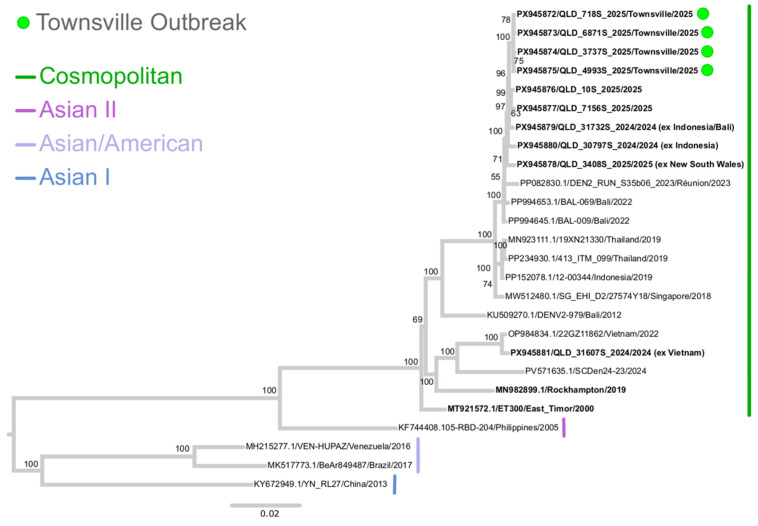
**DENV-2 Phylogenetic Tree.** Midpoint rooted maximum likelihood (ML) phylogenetic tree inferred from 26 dengue virus type 2 (DENV-2) complete coding region sequences (10,176 nucleotides) using the GTR+F+G4 nucleotide substitution model. The multiple sequence alignment was performed using the Multiple Alignment using Fast Fourier Transform (MAFFT) program [21], version 7.490 within Geneious Prime software, version 2026.0.2. The phylogenetic tree was constructed using IQTree version 3.0.1 [22] with bootstrap support estimated from 1000 replicates and percentage values are shown on key nodes. DENV-2 sequences from Queensland (QLD) patients are highlighted in bold font and sequences from locally transmitted QLD DENV-2 cases from the Townsville 2024/2025 outbreak (GenBank accession numbers PX945872 to PX945875) are further highlighted by a green dot. Respective DENV-2 genotypes (Cosmopolitan, Asian II, Asian/American and Asian I) are also indicated.

All specimens from confirmed cases were sent to Public Health Virology (PHV), Public and Environmental Health Reference Laboratories (PEHRL) for DENV laboratory investigations [20]. DENV-2 was detected on reverse transcriptase real-time polymerase chain reaction (RT-rtPCR) in eleven of the twelve confirmed cases. DENV-2 viral genomic nucleic acid sequencing and phylogenetic analysis was undertaken on four patient samples, two patients from North Ward and two patients from South Townsville. DENV-2 near whole genome sequences (QLD_718S_2025, QLD_6871S_2025, QLD_3737S_2025, and QLD_4993S_2025 with GenBank accession numbers PX945872 to PX945875, respectively) were obtained from these four patients, and DENV-2 isolates were obtained via virus culture from patients with sequences QLD_718S_2025 and QLD_4993S_2025. Phylogenetic analysis demonstrated that all four patient sequences grouped closely together in the phylogenetic tree (Figure 3) within the Cosmopolitan genotype, and with the exception of ambiguous bases in sequence QLD_3737S_2025, shared high nucleotide identity of >99.9%.

The TPHU medical entomology team collected adult *Ae aegypti* mosquitoes using standard BG-Sentinel 2 Mosquito Traps (Biogents, Regensburg, Germany) to determine *Wolbachia* frequency. Trapping occurred in December 2024 (South Townsville) and January 2025 (North Ward) over seven days. Collections were sent to the World Mosquito Program, Victoria, Australia, and screened for *Wolbachia* using standard PCR protocols [23]. One hundred and thirty-one mosquitoes were trapped, and 126 mosquitoes carried *w*Mel *Wolbachia* (96.1%). This is consistent with the prevalence of *Wolbachia*-carrying mosquitoes since the program was established in the Townsville region.

### 3.3. Public Health Response

TPHU managed the outbreak response, with a focus on health education and local vector control. Each probable and confirmed case was interviewed as per state process and guidelines immediately upon receipt of the unit being notified. They were provided education on vector control measures such as spraying insecticide inside their home and removing mosquito breeding sites, mosquito bite prevention, and signs and symptoms requiring urgent medical attention. Furthermore, the TPHU entomology team performed vector control measures including interior and exterior residual spraying and breeding site removal or treatment at the case and accessible surrounding residences, as per state guidelines.

Whilst active case finding was not a predominant component of TPHU’s outbreak response, the public health clinicians advised that people living and visiting in the case’s households should consider testing if symptomatic. Following these alerts, three additional probable cases and one confirmed case were identified.

As per protocol, following a notification of a confirmed case of locally acquired dengue, TPHU released a media statement advising the public on ways to reduce the risk of mosquito bites and what to do if they are concerned regarding potential exposure and infection that may result in dengue. As more notifications of probable and confirmed cases from North Ward were received, TPHU declared an outbreak on 12 March 2025 and a second media statement was released notifying the public of the outbreak, promoting mosquito preventative activities at a population level and advising symptomatic people to get tested. TPHU also alerted general practitioners and hospital clinicians to advise on appropriate testing and notification procedures.

The TPHU entomology team visited 58 houses during the outbreak. Nineteen households were treated with an interior residual spray, and eight with an exterior harborage spray with Biflex Aquamax (FMC Corporation) using a 100 g/L formulation of bifenthrin. A total of 71 containers were either removed, tipped out or treated with Prolink *S*-methoprene insect growth regulator pellets (Pacific Biologics, Kippa Ring, Queensland, Australia). Twenty-three of the 71 containers had *Ae aegypti*.

## 4. Discussion

Episodic outbreaks of dengue occurred in the north Queensland region almost annually prior to the implementation of the *Wolbachia*-infected mosquito vector [14,18]. The Townsville region had not had a local outbreak of dengue since 2020, largely attributed to the introduction of *Wolbachia*-infected mosquitoes with the *w*Mel strain, owing to the consistent presence and high incidence of these mosquitoes which has been shown to represent >90% of the mosquito population in Townsville since 2018 [16]. This is in contrast to other north Queensland DENV-susceptible regions such as the Torres Strait where *w*Mel *Wolbachia*-infected *Ae. aegypti* have not yet been introduced and DENV outbreaks continue to periodically occur. In 2024, prior to the DENV-2 2024–2025 Townsville outbreak, a local DENV-3 outbreak occurred in the Torres Strait, implicating *Ae. albopictus* which resulted in eight confirmed cases [24].

The 2024–2025 outbreak was the second autochthonous DENV outbreak in Townsville and resulted in the highest number of locally acquired mainland cases within north Queensland since deployment of the *Wolbachia* control strategy, highlighting concerns for ongoing DENV outbreak risk factors for the region, particularly during wet seasons and periods of above-average rainfall conditions. This includes temperature and other environmental factors, human behavior, and the consideration of associated DENV mosquito vector species given the ongoing risk of incursions and establishment of *Ae. albopictus* mosquito populations on the mainland.

Townsville had significant rainfall during the outbreak period, making the environmental conditions ideal for mosquito breeding [25]. Visits to the homes showed numerous potential mosquito breeding sites, but without a comparator, we cannot definitively determine whether there were more mosquito breeding sites than usual and if the higher rainfall contributed to the number of mosquito breeding sites. Whilst the percentage of *Wolbachia*-infected mosquitoes detected remained above 95% during the outbreak, the number of *Wolbachia*-uninfected mosquitoes is expected to have been proportionately higher.

Whilst a high percentage (96.1%) of *w*Mel *Wolbachia*-infected mosquitoes was detected from a small cohort of 131 mosquitoes collected in December 2024 and January 2025, the actual number of *Wolbachia*-uninfected *Ae. aegypti* present in the Townsville region potentially contributing to the dengue outbreak is unknown. Exacerbated rainfall or other environmental conditions during the outbreak period and beyond the mosquito cohort collection dates in December 2024 and January 2025 could have increased numbers of *Wolbachia*-uninfected *Ae. Aegypti*. Actual numbers and the rate of *w*Mel *Wolbachia*-infected mosquito reproduction or integration into Townsville *Ae. aegypti* populations during the same outbreak period are also unknown and pose interesting questions regarding the effectiveness of the control strategy during periods of unusually increased rainfall and/or other atypical environmental conditions.

Cellular *Wolbachia* density is known to decrease with high temperatures [26,27]; however, Townsville did not have any heat waves or experience average temperatures in excess of the historical mean before or during this period. Therefore, the temperature was not considered to be a factor in this outbreak [19].

With the reduction in locally acquired cases since the introduction of the *Wolbachia* mosquito control strategy, there have been fewer media and health promotion activities, which may have reduced the community’s awareness of dengue as a potentially serious disease, and their knowledge about an individual’s role in dengue prevention. Prior to the *Wolbachia* program, there were prominent health promotion campaigns about dengue prevention in north Queensland, which were successful in enhancing community members’ knowledge, attitudes and behaviors [28]. Future research into the north Queensland community’s knowledge, attitudes and behaviors about dengue and dengue prevention would help public health units understand what health promotion activities are needed in the lead up to and during the wet season.

Public awareness regarding the risk of dengue infection during overseas travel, particularly in dengue endemic areas, should also be emphasized and included in ongoing community education and alerts. Many DENV infections are asymptomatic [29], and travelers who are better informed about the risks of DENV exposure and mosquito bite prevention may further contribute to reducing DENV incursions into Australia. Historically, the identification of specific index cases of local DENV outbreaks in Australia is rare and no such case was identified during the Townsville 2024–2025 outbreak. To attempt to identify the likely source of the Townsville DENV-2 2024–2025 outbreak and genomically characterize the virus strain involved, whole-genome sequencing and phylogenetic analysis were performed. Townsville patient-derived DENV-2 sequences obtained from South Townsville (*n* = 2) and North Ward (*n* = 2) suburbs during the outbreak between early January 2025 and mid-March 2025 demonstrated a high degree of nucleotide identity, indicating transmission of the same DENV-2 strain belonging to the Cosmopolitan genotype. In comparison with globally publicly available DENV-2 sequences, the Townsville 2024–2025 outbreak strain was most closely related to recent DENV-2 sequences (GenBank accession numbers PX945876 to PX945880) obtained from other Queensland and New South Wales patients, some of which had known travel history to Bali/Indonesia or had potentially been in contact with cases who had traveled to that region or elsewhere in Southeast Asia (Figure 3). In the current absence of more closely related publicly available sequence data, further contextualization of the phylogenetic tree and more specific identification of the likely geographical origin of the Townsville 2024–2025 DENV-2 outbreak was limited.

There were also a few limitations regarding identifying cases during this outbreak. During late January and February, there was a significant rain event which may have limited access to healthcare, either due to power outages, flooding, or road closures that may have impacted individuals’ ability to seek medical attention. The messaging about the dengue outbreak may not have been received well by the community due to their competing priorities with the weather event, and there may have been a delay in community awareness, leading to symptomatic patients not seeking testing within appropriate timeframes.

Another limitation of this outbreak report was understanding any changes in the intrinsic and extrinsic factors of the *w*Mel *Wolbachia*-infected mosquito vector. The genotype of the species and strains of the mosquito vector have been known to influence the level of susceptibility and resistance to *Wolbachia* infection and propagation [30]. Published literature on the genotypic changes in *Ae. aegypti* in Townsville and surrounding regions are limited—warranting future research to understand genetic changes in the vector influencing *Wolbachia* density and susceptibility to DENV replication compared to those released in 2014–2017. Extrinsic factors other than that mentioned above, such as the mosquito vector’s diet [30] or the general use of insecticides, are not thought to have contributed to this outbreak. Further research and surveillance of vector competence, *Wolbachia* density, and insecticide susceptibility would provide an evidence base to understand the performance of the prevention strategy compared to implementation in 2014–2017.

This outbreak has demonstrated the importance of TPHU and other north Queensland public health units in continuing to investigate probable and confirmed dengue notifications, and to monitor the percentage of *Wolbachia*-infected mosquitoes in the region together with continued DENV surveillance and case confirmation supported by Queensland Health laboratory investigations. TPHU will review previous health promotion strategies and reinforce health promotion on dengue prevention in the Townsville community.

## Data Availability

Rainfall and temperature data presented in the study are openly available in the Australian Government’s Bureau of Meteorology Climate Data Online portal at https://www.bom.gov.au/climate/data/ (accessed on 18 December 2025). Data relating to screening of *Wolbachia* in trapped *Aedes aegypti* mosquitoes was obtained from the World Mosquito Program and therefore availability is restricted. Permission to sought for these data need to be made to contact@worldmosquitoprogram.org. Clinical patient data of the notified cases presented in this article are not readily available due to privacy restrictions. Requests to access the datasets should be directed to the corresponding author. All virus sequences are available in the National Center for Biotechnology Information GenBank database at https://www.ncbi.nlm.nih.gov/genbank/ (accessed on 18 December 2025).

## Abbreviation

The following abbreviation is used in this manuscript:

TPHU	Townsville Public Health Unit

## Appendix A

**Table A1:** Line list in order of symptom onset.

Case	Suburb	Gender	Date of Symptom Onset	Date of First Specimen Collection	Notification Date (Days Since Symptom Onset)	Test Result	Status
1	North Ward	Male	24 December 2024	3 January 2025	7 January 2025 (15 days)	Flavivirus IgM +/IgG +; Flavivirus unspecified	Probable—epidemiological link
2	North Ward	Male	6 January 2025	7 January 2025	13 January 2025 (8 days)	Dengue NS1 + PCR: Dengue 2	Confirmed
3	South Townsville	Male	31 January 2025	7 February 2025	11 February 2025 (12 days)	Dengue NS1 + Flavivirus IgM +/IgG +; Flavivirus unspecified	Confirmed
4	South Townsville	Male	4 February 2025	11 February 2025	13 February 2025 (10 days)	Flavivirus IgM +/IgG +; Flavivirus unspecified PCR: Dengue 2	Confirmed
5	South Townsville	Female	7 February 2025	11 February 2025	18 February 2025 (12 days)	Flavivirus IgM +/IgG +; Flavivirus unspecified Dengue IgM +/IgG + PCR: Dengue 2	Confirmed
6	South Townsville	Male	17 February 2025	20 February 2025	25 February 2025 (9 days)	PCR: Dengue 2	Confirmed
7	North Ward	Male	29 January 2025	-	11 March 2025 (42 days)	Nil	Probable—household epidemiological-link
8	North Ward	Male	20 February 2025	27 February 2025	3 March 2025 (15 days)	Flavivirus IgM +/IgG +; Flavivirus unspecified	Probable—epidemiological-link
9	North Ward	Male	21 February 2025	3 March 2025	11 March 2025 (19 days)	Flavivirus IgM +/IgG +; Flavivirus unspecified	Probable—epidemiological-link
10	North Ward	Male	12 March 2025	13 March 2025	17 March 2025 (6 days)	PCR: Dengue 2	Confirmed
11	North Ward	Female	3 March 2025	5 March 2025	11 March 2025 (9 days)	Flavivirus IgM +/IgG + PCR: Dengue 2	Confirmed
12	North Ward	Male	13 March 2025	14 March 2025	17 March 2025 (5 days)	PCR: Dengue 2	Confirmed
13	North Ward	Male	10 March 2025	12 March 2025	14 March 2025 (5 days)	PCR: Dengue 2	Confirmed
14	North Ward	Female	15 March 2025	17 March 2025	20 March 2025 (6 days)	PCR: Dengue 2	Confirmed
15	North Ward	Male	20 March 2025	26 March 2025	27 March 2025 (8 days)	Flavivirus IgM +/IgG +; Dengue unspecified	Confirmed
16	North Ward	Male	17 March 2025	21 March 2025	14 April 2025 (29 days)	PCR: Dengue 2	Confirmed

## Appendix B

### Dengue Virus Case Definitions, as Endorsed by the Queensland Health Guideline for Public Health Units [9], Onset Between December 2024 and March 2025

In accordance with the Queensland Health guidelines for public health units, cases were defined as follows:

Confirmed case

• Laboratory definitive evidence and clinical evidence
  ○ Laboratory definitive evidence
    ▪ Isolation of dengue virus OR
    ▪ Detection of dengue virus by nucleic acid testing OR
    ▪ Detection of non-structural protein 1 (NS1) antigen in blood by EIA OR
    ▪ IgG seroconversion or a significant increase in antibody level or a fourfold or greater rise in titre to dengue virus, proven by neutralization or another specific test OR
    ▪ Detection of dengue virus-specific IgM in cerebrospinal fluid, in the absence of IgM to Murray Valley encephalitis, West Nile virus/Kunjin, or Japanese encephalitis viruses.
  ○ Clinical evidence
    ▪ A clinically compatible illness (e.g., Fever, headache, arthralgia, myalgia, rash, nausea/vomiting).

Probable case

• Laboratory suggestive evidence and clinical evidence and epidemiological evidence OR Clinical evidence and household epidemiological evidence
• Laboratory suggestive evidence
  ○ Detection of NS1 antigen in blood by a rapid antigen test OR
  ○ Detection of dengue-virus-specific IgM in blood
• Epidemiological evidence
  ○ Exposure, between 3 and 14 days prior to onset, in either a country with known dengue OR a dengue-receptive area in Australia where a locally acquired or imported case has been documented with onset within a month.
• Household epidemiological evidence
  ○ Living in the same house as a locally acquired case in a dengue-receptive area of Australia within a month of the onset of the case AND
  ○ At least one case in the chain of epidemiologically linked laboratory-confirmed cases (which may involve many cases).
